# Effects of Adding Treadmill Training to Conventional Physiotherapy on Lower-Limb Thermal Asymmetry, Motor Function, and Walking Capacity in Patients with Chronic Stroke: A Randomized Controlled Trial

**DOI:** 10.3390/brainsci16080837

**Published:** 2026-08-06

**Authors:** Marta Gómez-Mateos, Luis Augusto Silva-Zendron, Andrea Calleja-Caballero, Vanesa Santos-Rodríguez, Beatriz María Bermejo-Gil, Fátima Pérez-Robledo

**Affiliations:** 1 Department of Nursing and Physiotherapy, Faculty of Nursing and Physiotherapy, Universidad de Salamanca, 37008 Salamanca, Spain; andreacallejac@usal.es (A.C.-C.); vanesasantosr@usal.es (V.S.-R.); beatriz.bermejo@usal.es (B.M.B.-G.); fatima_pr@usal.es (F.P.-R.); 2 Institute of Biomedical Research of Salamanca [IBSAL], 37007 Salamanca, Spain; 3 Department of Computer Science, Universidad de Salamanca, 37008 Salamanca, Spain; luisaugustos@usal.es

**Keywords:** stroke, thermography, physical therapy modalities, lower extremity, motor skills

## Abstract

**Highlights:**

**What are the main findings?**

- Rehabilitation was associated with reduced lower-limb thermal asymmetry in individuals with chronic stroke.
- Treadmill training added to conventional physiotherapy improved lower-limb motor function and walking capacity.

**What are the implications of the main findings?**

- Infrared thermography may provide additional information alongside conventional functional assessments.
- Thermographic and functional measures may capture different aspects of recovery following rehabilitation.

**Abstract:**

**Background/objectives**: Thermal asymmetries between the affected and unaffected sides of the body are common after stroke and can be detected using infrared thermography; however, their responsiveness to rehabilitation and its relationship with motor and functional performance remain unclear. This study investigated whether the addition of treadmill training to conventional physiotherapy modifies lower-limb thermal asymmetry and explored its relationship with functional outcomes in individuals with chronic stroke. **Methods**: A randomized controlled trial was conducted in 22 individuals with chronic stroke randomly assigned to either an intervention group [*n* = 12] or a control group [*n* = 10]. All participants received conventional physiotherapy twice weekly for 12 weeks, while the intervention group additionally performed treadmill training. Thermal asymmetry was assessed using infrared thermography, whereas motor function and walking capacity were evaluated using the Fugl–Meyer Assessment for the Lower Extremity and the Six-Minute Walk Test. **Results**: Both groups showed reduced thermal asymmetry after the intervention, with no significant between-group differences. Significant improvements in motor function (*p* = 0.016) and walking distance (*p* = 0.016) were observed only in the intervention group. Between-group analysis revealed greater motor improvement in the intervention group (*p* = 0.017). Baseline-adjusted analyses confirmed superior motor function and walking capacity outcomes in the intervention group. **Conclusions**: Infrared thermography detected changes in lower-limb thermal asymmetry following the 12-week physiotherapy program in individuals with chronic stroke, with no significant between-group differences. In contrast, treadmill training produced superior improvements in motor function and walking capacity. These differing patterns suggest that thermographic and functional assessments may provide complementary information regarding recovery after chronic stroke.

## 1. Introduction

Chronic stroke sequelae result in significant asymmetries between the affected and unaffected sides of the body [1]. These asymmetries are not only expressed at the functional level but are also associated with disruptions in peripheral physiological regulation, including alterations in autonomic activity, tissue perfusion, and metabolism [2,3]. Such disturbances may result in temperature differences between body sides, which have been described in the literature [4] and can be detected using infrared thermography [1,5]. However, these thermal asymmetries remain underexplored and may reflect underlying alterations in vascular regulatory mechanisms and functional status.

Infrared thermography [4,6,7] has emerged as a non-invasive, accessible, and clinically feasible tool for assessing skin temperature distribution and identifying thermal asymmetries, which may indirectly reflect peripheral processes. Although thermal asymmetries in the lower limbs following stroke have been described using thermography [4,8], their evolution in response to rehabilitation and their relationship with motor function and functional performance remain insufficiently explored.

These thermal asymmetries should not be interpreted merely as alterations in blood circulation, as peripheral physiological disturbances may also contribute to sensory and motor deficits [1]. In this context, skin temperature may act as an indirect indicator of these peripheral physiological processes, providing complementary information to that obtained through conventional functional assessments.

From an integrative perspective, thermal and locomotor alterations appear to interact bidirectionally. Impaired perfusion and metabolism dysfunction, reflected in altered skin temperature, may limit the functional capacity of the affected limb [9,10], promote altered movement patterns, and influence its role during locomotion [9]. Conversely, reduced neuromuscular activation of the paretic limb may further decrease local temperature [10], reinforcing these peripheral alterations. This relationship suggests that thermal asymmetry not only reflects peripheral physiological status but may also be associated with functional performance limitations, potentially acting as a sensitive marker of peripheral adaptations linked to reduced functional capacity [4].

In the chronic phase of stroke, where recovery tends to be slower [11,12], the persistence of these alterations represents an important clinical challenge. In addition to motor weakness, lower-limb spasticity frequently contributes to gait dysfunction. Proximal spasticity may impair hip and knee control, whereas distal spasticity commonly results in foot deformities, limiting limb advancement, reducing foot clearance, and promoting abnormal gait patterns [13,14]. These impairments compromise walking performance and reinforce the importance of comprehensive rehabilitation approaches. However, conventional clinical assessment primarily focuses on functional and motor outcomes, providing limited information about the peripheral physiological adaptations that accompany recovery. This highlights the need for sensitive tools capable of detecting physiological changes associated with rehabilitation. Accordingly, it is important to investigate whether rehabilitation interventions aimed at improving function are also capable of modifying these peripheral thermal alterations.

Among rehabilitation strategies, treadmill training has demonstrated consistent benefits on multiple functional outcomes [15]. Its task-specific and repetitive nature allows for the modulation of training volume and intensity, while the control of speed and load facilitates the application of sufficient intensity stimuli [16] to induce neuromuscular and peripheral adaptations [17].

Evidence suggests that moderate- to high-intensity treadmill training produces clinically meaningful improvements in motor function and functional performance in individuals with chronic stroke, outperforming low-intensity or overground interventions [18,19]. These benefits are thought to be mediated by enhanced muscle activation, improved neuromuscular coordination, and greater gait symmetry [20]. Previous treadmill studies have primarily focused on locomotor and functional outcomes, whereas the peripheral thermal response to this type of task-specific training has received considerably less attention [18,19]. Although the functional effects of treadmill training after stroke are relatively well established, it remains unclear whether these improvements are accompanied by measurable changes in peripheral thermal regulation. Evidence regarding the responsiveness of lower-limb thermal asymmetry to treadmill-based rehabilitation is scarce, and thermographic outcomes have rarely been integrated with conventional motor and walking assessments within controlled intervention studies. Addressing this gap may help determine whether thermography captures rehabilitation-induced changes that complement those identified through conventional functional outcomes.

Therefore, the aim of the present study was to analyze whether the addition of treadmill training to conventional physiotherapy over a 12-week period modifies lower-limb thermal asymmetry and to explore the relationship between thermographic measures, motor function, and functional performance, thereby contributing to a better understanding of the mechanisms underlying functional recovery in this population.

## 2. Materials and Methods

### 2.1. Study Design and Setting

A single-blind, parallel-group randomized controlled trial with a 1:1 allocation ratio was conducted at the Faculty of Nursing and Physiotherapy, University of Salamanca. This trial was reported in accordance with the CONSORT (Consolidated Standards of Reporting Trials) statement, and the intervention was described according to the TIDieR checklist.

The study received approval from the Bioethics Committee of the University of Salamanca (approval number: 1347) and was conducted in accordance with the Declaration of Helsinki. The trial was registered at ClinicalTrials.gov (https://clinicaltrials.gov/study/NCT07127861, accessed on 3 August 2026; identifier: NCT07127861, INV-2025-58) on 10 August 2025.

All participants were informed about the study objectives and procedures and provided written informed consent prior to participation. In addition, participants explicitly consented to the anonymous use and publication of their data in a scientific journal.

### 2.2. Sample Size

The sample size was calculated a priori using data from a previous study involving a comparable population of individuals with stroke [21]. Although lower-limb thermal asymmetry was defined as the primary outcome of the present study, no previous longitudinal studies were available reporting intervention-induced changes in thermographic asymmetry in individuals with chronic stroke. Consequently, it was not possible to obtain reliable estimates of the expected effect size or variability for thermographic outcomes.

Therefore, the Fugl–Meyer Assessment for the Lower Extremity (FMA-LE) [22] was selected as the reference outcome for sample size estimation. The FMA-LE is a validated and clinically relevant measure of lower-limb motor function for which variability estimates and clinically meaningful changes have been previously reported in comparable stroke populations.

As no previous intervention studies had reported effect size estimates for the original FMA-LE protocol (0–86), the sample size calculation was based on published estimates for the validated motor subscale of the FMA-LE (0–34), which represented the best available evidence at the time the study was designed.

A clinically relevant difference of 6 points was assumed according to previous literature reporting the minimal clinically important difference for lower-limb motor recovery after stroke [23]. The variance estimate (45.29 points^2^) was obtained from previously published FMA-LE data in a comparable stroke population (SD = 6.73 points). Assuming a two-sided significance level of 0.05, a statistical power of 80%, and an anticipated dropout rate of 25%, the minimum required sample size was estimated at 22 participants. Therefore, 22 participants were recruited.

### 2.3. Participants

**Figure 1:**
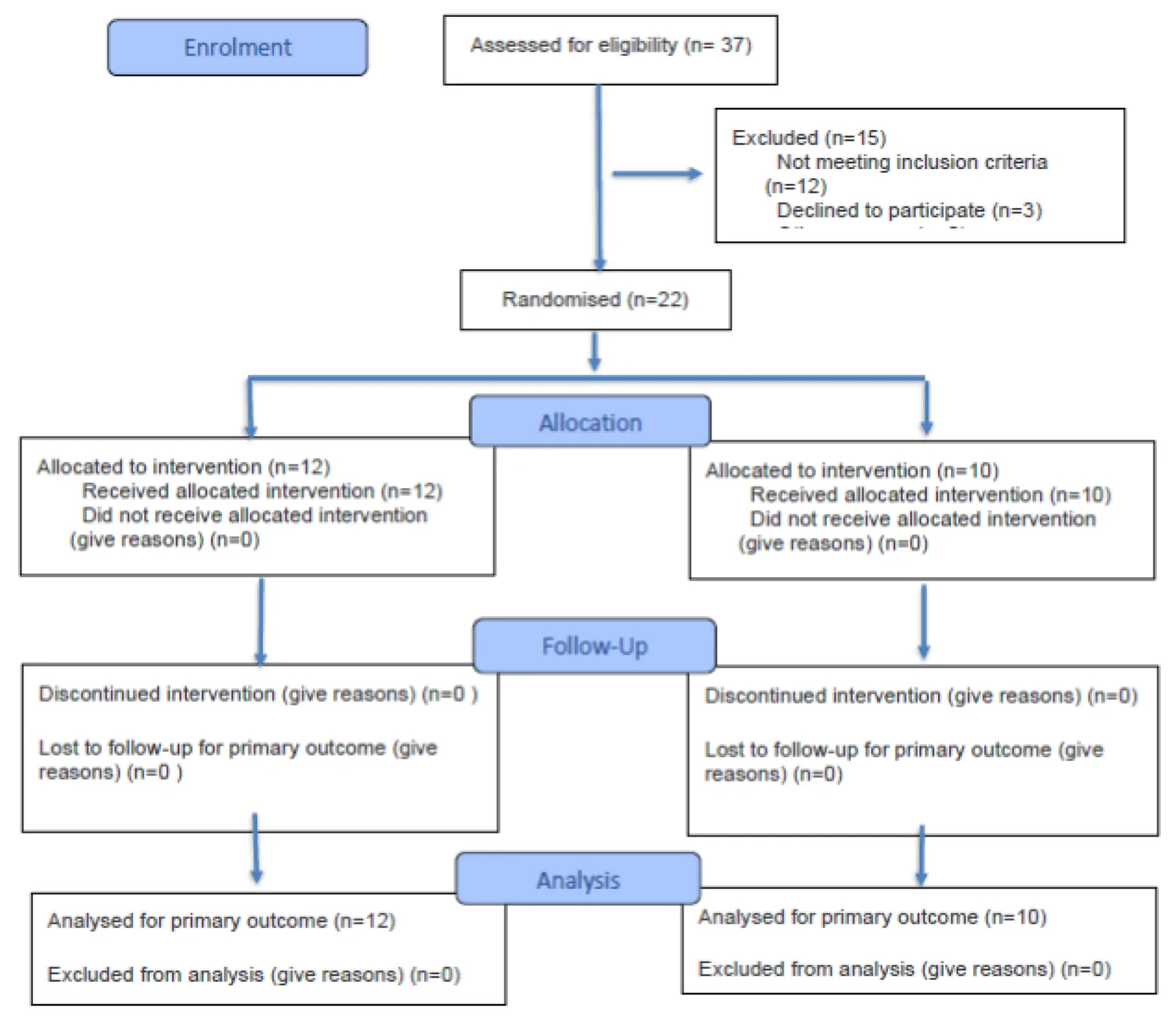
CONSORT flow diagram of participant recruitment, allocation, follow-up, and analysis.

A total of 37 individuals with stroke were screened for eligibility at a local acquired brain injury association. Of these, 22 met the inclusion criteria and were randomly allocated to study groups, while the remaining individuals were excluded for not meeting the eligibility criteria or declining participation (Figure 1) [24].

Inclusion criteria were age between 18 and 80 years, diagnosis of chronic stroke (>6 months), presence of motor or sensory impairments predominantly affecting one side of the body, and ability to walk independently with or without assistive devices. Exclusion criteria included cognitive impairments preventing understanding of the instructions, inability to safely use a treadmill, severe heart failure, uncontrolled arterial hypertension, and the presence of active inflammatory or infectious conditions at the time of assessment.

### 2.4. Randomization and Blinding

Eligible participants were randomly allocated to either the intervention or control group using an envelope-based randomization procedure. Opaque, consecutively numbered envelopes containing a unique identification number were prepared in advance and mixed to generate a random sequence. After baseline assessment, participants selected and opened an envelope in the presence of a researcher not involved in outcome assessment, with group assignments determined by odd or even numbering. Outcome assessors were blinded to group assignments. The randomization list was securely stored by the investigator responsible for allocation and was not accessible to assessors at any point during the study, ensuring appropriate allocation concealment.

### 2.5. Outcome Measures

The primary outcome was thermal asymmetry of the lower limbs. Secondary outcomes included FMA-LE scores and 6MWT distance. All outcomes were assessed at baseline and after 12 weeks.

Accordingly, the primary hypothesis was that the addition of treadmill training to conventional physiotherapy would result in a greater reduction in lower-limb thermal asymmetry compared with conventional physiotherapy alone. The secondary hypothesis was that the addition of treadmill training would result in greater improvements in lower-extremity motor function and walking capacity.

Lower-limb function was assessed using the expanded version of the FMA-LE a reliable tool for evaluating post-stroke sensorimotor impairment. Assessments were conducted following standardized procedures using the Spanish version of the FMA-LE protocol translated by the Hospital Militar Central and the Universidad Nacional de Colombia from the original University of Gothenburg protocol [22], with scores obtained through direct observation of task performance.

The validated motor domain of the FMA-LE evaluates lower-limb motor function, with scores ranging from 0 to 34 points. In the present study, an expanded version was used that additionally includes the sensory function, passive joint motion, and joint pain domains, yielding a total score ranging from 0 to 86 points. The original validated motor domain evaluates voluntary motor control through hierarchically organized tasks, ranging from reflex activity to movements within and out of synergy patterns, as well as coordination and speed, with scores ranging from 0 to 34. In the present study, an expanded version was used that additionally includes the sensory function, passive joint motion, and joint pain domains, resulting in a total score ranging from 0 to 86. The sensory domain assesses light touch and proprioception, while the passive joint motion and joint pain domains evaluate joint range of motion and pain response during passive movement. Each component is rated on a 3-point ordinal scale (0–2), where higher scores indicate better motor function and lower levels of impairment.

Functional walking endurance was assessed using the Six-Minute Walk Test (6MWT) [20], a widely used and reliable measure of walking performance in individuals with stroke [25]. The test was conducted along a flat, straight corridor, where participants were instructed to walk as far as possible at their maximum safe pace for six minutes. Standardized instructions were provided prior to the test, and encouragement was delivered at regular intervals using predefined phrases in accordance with established guidelines [26]. Walking aids were permitted, but no physical assistance was allowed. If participants experienced fatigue or discomfort, they were allowed to stop and rest; however, the timing protocol continued uninterrupted, and participants resumed walking once able. The total distance covered (in meters) was recorded, with greater distances indicating better walking capacity.

Lower-limb thermal asymmetry was the primary outcome and was evaluated using infrared thermography following a standardized protocol consistent with the Thermographic Imaging in Sports and Exercise Medicine (TISEM) consensus guidelines to ensure clinical applicability and reproducibility [27].

Thermal images were acquired using a FLIR C5 infrared camera, which offers a thermal resolution of 160 × 120 pixels, a frame rate of 8.7 Hz, a spectral range of 8–14 µm and a noise equivalent temperature difference < 70 mK, sufficient for clinical thermographic evaluations [28]. All measurements were conducted in a fully enclosed room under controlled environmental conditions, with ambient temperature and relative humidity continuously monitored throughout data acquisition. Participants underwent a 15 min acclimatization period prior to imaging, remaining seated, barefoot, and without physical contact with the lower limbs. Participants were instructed to avoid physical exercise, exposure to heat or cold, topical products, caffeine, alcohol, and smoking for at least two hours before assessment. Clothing was standardized (swimsuit/shorts) to ensure full visualization of thighs, legs, and feet. Participants were also instructed to report symptoms of fever or infection, which could affect thermographic stability and lead to exclusion of the session [2].

For standing thermograms, the infrared camera was mounted on a tripod positioned 2 m from the participant and set at a height of 1 m, while 0.5 m from the wall, to provide a uniform background. For plantar thermograms, participants were placed in the supine position, and the camera was positioned 0.5 m from the feet at the same height. Images were acquired in a standardized sequence: anterior lower limbs, posterior lower limbs, and plantar aspects.

Regions of Interest (ROIs) were selected based on previous scientific literature [29,30]. Anterior ROIs included the thigh and leg bilaterally. Posterior ROIs included proximal and distal regions of both legs. Plantar temperature was recorded at two predefined points located at the medial and lateral aspects of the foot. Thermal asymmetry was calculated as the absolute temperature difference between the affected and unaffected limbs. A temperature difference ≥ 0.5 °C was considered clinically meaningful [26,31].

### 2.6. Thermal Image Analysis

Thermogram processing was performed offline using specialized radiometric analysis software (FLIR Ignite Tools). Each captured image (radiometric JPEG format) contained a matrix of absolute temperature data for every pixel (19,200 measurement points), allowing for precise post hoc parameter readjustment.

Prior to data extraction, the quality of each thermogram was verified to ensure the absence of motion artifacts or thermal blurring. Global corrections were applied to the object parameters within the software. The emissivity was fixed at 0.98, the accepted standard value for human skin regardless of pigmentation. The reflected apparent temperature was individually adjusted according to the ambient temperature recorded during each session to compensate for environmentally infrared radiation reflected on the patient’s skin.

The definition of measurement areas was performed manually using polygon and ellipse tools on the thermal images, guided by the visual image edge overlay (MSX technology). Using real-time processing algorithms, the system extracts high-contrast details (edges, contours, textures) from the visual image and superimposes (“embosses”) them onto the thermal image. This allowed for the delimitation of bilateral ROIs following standardized anatomical references to avoid the “thermal edge effect” (temperature drop at the curvature of the limb).

For each ROI, the maximum, minimum, and average temperature values were calculated. However, for the main comparative analysis, the average temperature was selected as a more representative indicator of the entire muscle group and is more robust in the face of isolated hot spots (noise or superficial vascularization).

Image analysis was conducted by a single researcher, blinded to the patients’ group allocation and supervised by the physiotherapists, following the same standardized anatomical references and segmentation procedure for all thermograms in order to minimize variability during ROI segmentation.

### 2.7. Intervention

The intervention was conducted over a 12-week period. All participants received conventional neurological physiotherapy twice weekly, each lasting 40 min, with a minimum interval of 48 h between sessions. A total of 24 sessions (960 min) were therefore completed by each participant.

The conventional physiotherapy program was individualized and targeted sensorimotor impairments commonly observed after stroke, with progressive adjustments in exercise intensity and task complexity according to individual performance and tolerance. It included exercises aimed at improving balance, lower-limb muscle strength, coordination, gait performance, and functional task-oriented activities. Exercises included postural alignment reeducation, reactive and anticipatory balance tasks, closed- and open-chain strengthening, functional mobility training and overground gait practice.

Participants assigned to the control group performed only the conventional physiotherapy program. Those in the IG completed the same program and additionally performed treadmill gait training, consisting of two weekly sessions of 15 min each, for a total of 24 supplementary sessions (360 min) of specific treadmill training.

Following each physiotherapy session, intervention-group participants underwent a standardized 10 min rest period before starting the treadmill program.

Training sessions were conducted on a SALTER RS-10 treadmill at 0% incline. Sessions began at the lowest available speed and were progressively increased until participants reached an exertion level corresponding to 14 points on the modified Borg Rating of Perceived Exertion Scale [32]. Progression based on perceived exertion has been shown to be feasible and safe in stroke survivors [33]. All participants used a safety harness without body-weight support and were allowed to hold the handrails to prevent falls and increase confidence during training, in accordance with safety guidelines commonly used in treadmill-based stroke interventions [28]. Sessions were continuously supervised by a physiotherapist, who ensured correct positioning, monitored safety and stability throughout the protocol, and adjusted treadmill speed when necessary to maintain the target effort level. Verbal cues were used to optimize temporal gait parameters, such as biomechanical parameters, step length, and walking speed.

### 2.8. Adverse Events

Adverse events were monitored systematically throughout the intervention period. They were defined as any undesirable experience associated with the intervention, including musculoskeletal pain, fatigue beyond expected levels, cardiovascular symptoms, or any incident requiring interruption of the session. Participants were asked to report any symptoms during and after each session, and physiotherapists actively observed and recorded any incidents occurring during sessions.

### 2.9. Statistical Analysis

Statistical analyses were performed using IBM SPSS Statistics (version 30). Continuous variables related to motor function, functional walking capacity, and regional skin temperatures were summarized using means and standard deviations for each study group. Thermal asymmetries between the affected and unaffected limbs were also calculated to evaluate baseline thermal symmetry and its evolution following the intervention.

Continuous variables are presented as mean ± standard deviation to facilitate clinical interpretation and comparison with previous literature.

Given the small sample size and the lack of normality in several distributions, non-parametric tests were used to examine intervention-induced changes. Within-group comparisons between pre- and post-intervention measurements were performed using the Wilcoxon signed-rank test, allowing for assessment of changes in FMA-LE scores, 6MWT performance, regional temperatures, and thermal asymmetries. Z statistics and associated *p*-values were reported for each variable.

Between-group differences in the magnitude of change were analyzed using the Mann–Whitney U test, comparing absolute pre–post change scores for each variable. This analysis enabled the identification of differences in temperature increases across anatomical regions and in the progression of thermal symmetry.

Additionally, analysis of covariance (ANCOVA) was performed to compare post-intervention outcomes between groups while adjusting for baseline values. Separate ANCOVA models were constructed for FMA-LE, 6MWT, and thermal asymmetry variables, with post-intervention scores entered as dependent variables, group allocation as the fixed factor, and the corresponding baseline value as a covariate. Prior to conducting ANCOVA, the assumption of homogeneity of regression slopes was evaluated by testing the interaction between group and baseline values. As this assumption was met for all analyzed outcomes, adjusted group comparisons were considered valid. Partial eta-squared (ηp^2^) was calculated as a measure of effect size and interpreted as small (0.01), medium (0.06), or large (0.14).

To explore the relationship between physiological and functional adaptations, change scores were calculated for thermal asymmetry, FMA-LE, and 6MWT (post-intervention minus baseline). Associations between changes in thermal asymmetry and changes in FMA-LE and 6MWT were examined using Spearman’s rank correlation coefficient because of the small sample size and the non-normal distribution of several variables.

Effect sizes were calculated using Rosenthal’s correlation coefficient (r = Z/√N), where Z represents the standardized test statistic and N the number of observations, to facilitate interpretation of the magnitude of observed effects. Effect sizes were interpreted as small (r = 0.10), medium (r = 0.30), and large (r ≥ 0.50).

Mean changes and corresponding 95% confidence intervals (95% CI) were additionally calculated to facilitate interpretation of the magnitude and precision of treatment effects.

Analyses were conducted using the valid data available for each variable, with no imputation of missing values. Statistical significance was set at *p* < 0.05.

## 3. Results

### 3.1. Baseline Characteristics

A total of 22 participants were included, distributed into the intervention group (IG) (*n* = 12) and the control group (CG) (*n* = 10). All randomized participants completed the intervention and post-intervention assessments.

Baseline functional characteristics are presented in Table 1. Baseline lower-limb motor function was comparable between groups, with mean FMA-LE scores of 73.6 points in the IG and 67.2 points in the CG. However, a significant baseline difference was observed in functional walking capacity, with the IG demonstrating higher 6MWT performance (373.5 ± 115.8 m) than the CG (222.6 ± 131.9 m; *p* = 0.014). No significant differences were observed between groups for the remaining baseline variables, indicating the overall homogeneity of the sample.

### 3.2. Lower-Limb Motor Function (FMA-LE)

Lower-limb motor function showed different patterns depending on group allocation. When considering the entire sample, no statistically significant differences were observed between baseline and post-intervention FMA-LE scores (U = 48.0; Z = −0.871; *p* = 0.384), with mean values remaining stable (70.68 ± 11.39 vs. 71.09 ± 11.80).

Within-group analysis revealed a significant improvement in lower-limb motor function in the IG (Z = −2.401; *p* = 0.016), corresponding to a large effect size (r = 0.69) and a mean increase of 1.0 point (95% CI 0.34–1.66). No significant changes were observed in the CG (Z = −0.816; *p* = 0.414), with a small-to-moderate effect size (r = 0.26) and a mean change of –0.3 points (95% CI −1.26 to 0.66).

Between-group comparison of change scores showed statistically significant differences in the magnitude of improvement (Z = −2.388; *p* = 0.017; r = 0.51). The mean between-group difference was 1.3 points in favor of the IG (95% CI 0.24–2.36; *p* = 0.019).

### 3.3. Six-Minute Walk Test (6MWT)

In the pooled sample, the distance covered during the 6MWT increased significantly after the intervention period (Z = −1.979; *p* = 0.048), with a mean improvement of 74.7 m. This change was accompanied by a moderate effect size (r = 0.42).

Within-group analysis showed a significant improvement in the IG (Z = −2.401; *p* = 0.016; r = 0.69), corresponding to a large effect size, with walking distance increasing from 373.5 ± 115.8 m at baseline to 437.7 ± 104.4 m post-intervention, representing a mean gain of 64.2 m (95% CI 23.2–105.1 m). In contrast, the CG showed a mean change of –6.4 m and did not show significant changes in 6MWT performance (Z = −0.534; *p* = 0.594; r = 0.17), with similar values recorded at baseline (222.6 ± 131.9 m) and post-intervention (216.2 ± 131.6 m).

The between-group difference in change scores was 70.6 m in favor of the IG (95% CI 21.7–119.5 m; *p* = 0.007).

### 3.4. Regional Skin Temperature

Thermographic analysis demonstrated a consistent increase in skin temperature across all lower-limb regions and in both groups following the intervention. Overall, post-intervention temperatures converged toward values close to 31 °C, with mean increases ranging from approximately 2 °C to 4 °C depending on anatomical region and group (Table 2). These increases were statistically significant in all anatomical regions for both the affected and non-affected limbs in both groups (all *p* < 0.05).

At the anterior surface, both distal and proximal regions showed homogeneous temperature increases. In the IG, pre–post increases ranged from 2.1 °C to 3.0 °C, while the CG exhibited slightly larger increases in some regions, reaching up to 3.5 °C. Final temperatures ranged between 31.0 °C and 31.6 °C in both groups.

At the foot level, thermal increases were also consistent. The external foot region showed smaller increases of approximately 2 °C in both groups, whereas the internal foot region demonstrated more pronounced increases, ranging from 1.7 °C to 3.0 °C. Despite these differences, final temperatures were comparable between groups.

Posterior regions followed a similar pattern, with both distal and proximal areas exhibiting increases between 2 °C and 3 °C in both groups, reaching final temperatures ranging from 29.1 °C to 31.7 °C.

Overall, the pattern of temperature increase was comparable between groups, indicating that absolute skin temperature increased following rehabilitation irrespective of the intervention received. Changes in thermal asymmetry between the affected and non-affected limbs are presented separately in the following section, as this constituted the primary thermographic outcome of the study.

### 3.5. Thermal Asymmetry Pre–Post Intervention

**Figure 2:**
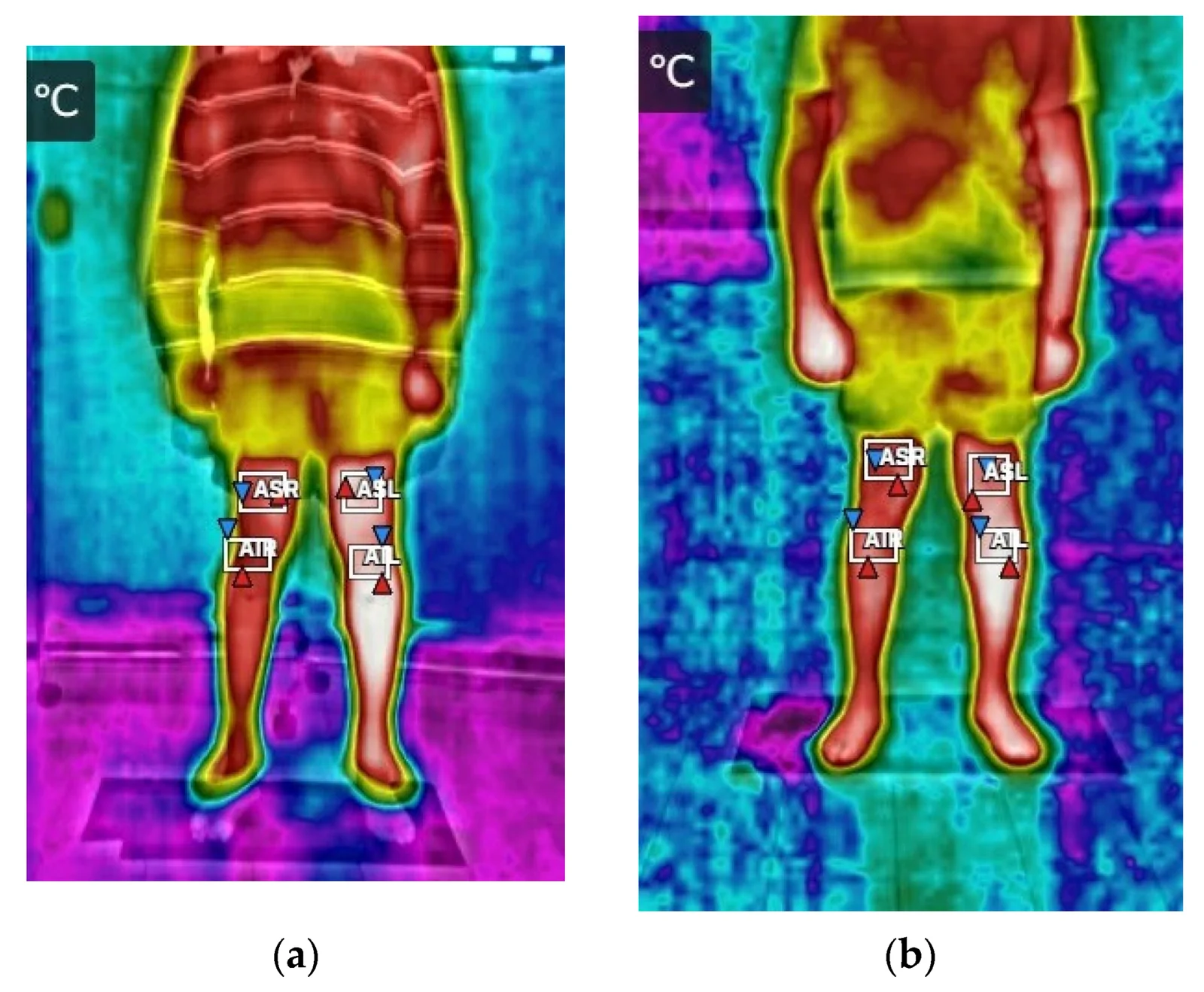
(**a**) Pre-intervention thermographic image showing anterior lower-limb temperature distribution. (**b**) Post-intervention thermographic image showing anterior lower-limb temperature distribution. ASR, anterosuperior right; ASL, anterosuperior left; AIR, anteronferior right; and AIL, anteroinferior left.

**Figure 3:**
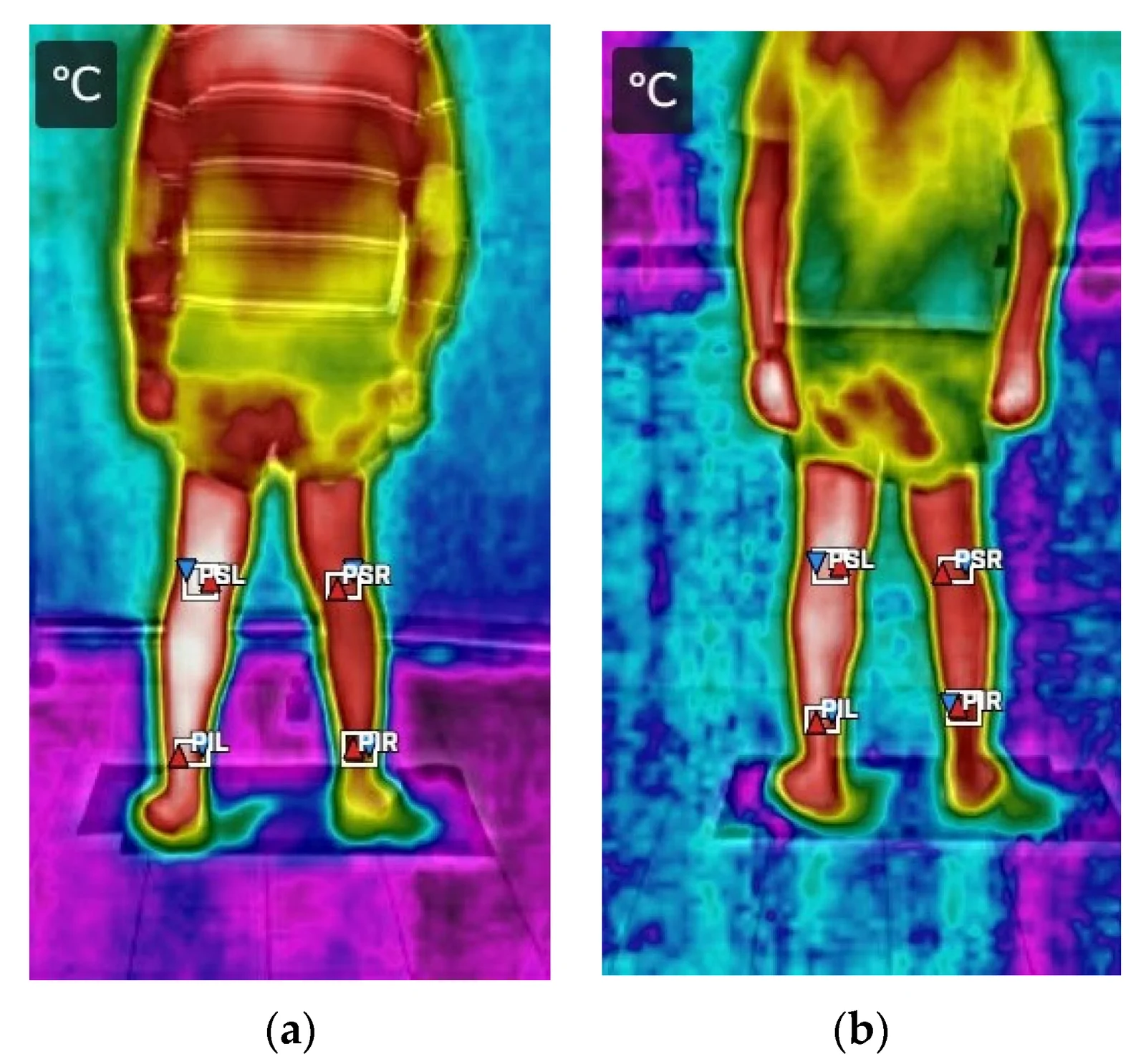
(**a**) Pre-intervention thermographic image showing posterior lower-limb temperature distribution. (**b**) Post-intervention thermographic image showing posterior lower-limb temperature distribution. PSR, posterosuperior right; PSL, posterosuperior left; PIR, posteroinferior right; and PIL, posteroinferior left.

**Figure 4:**
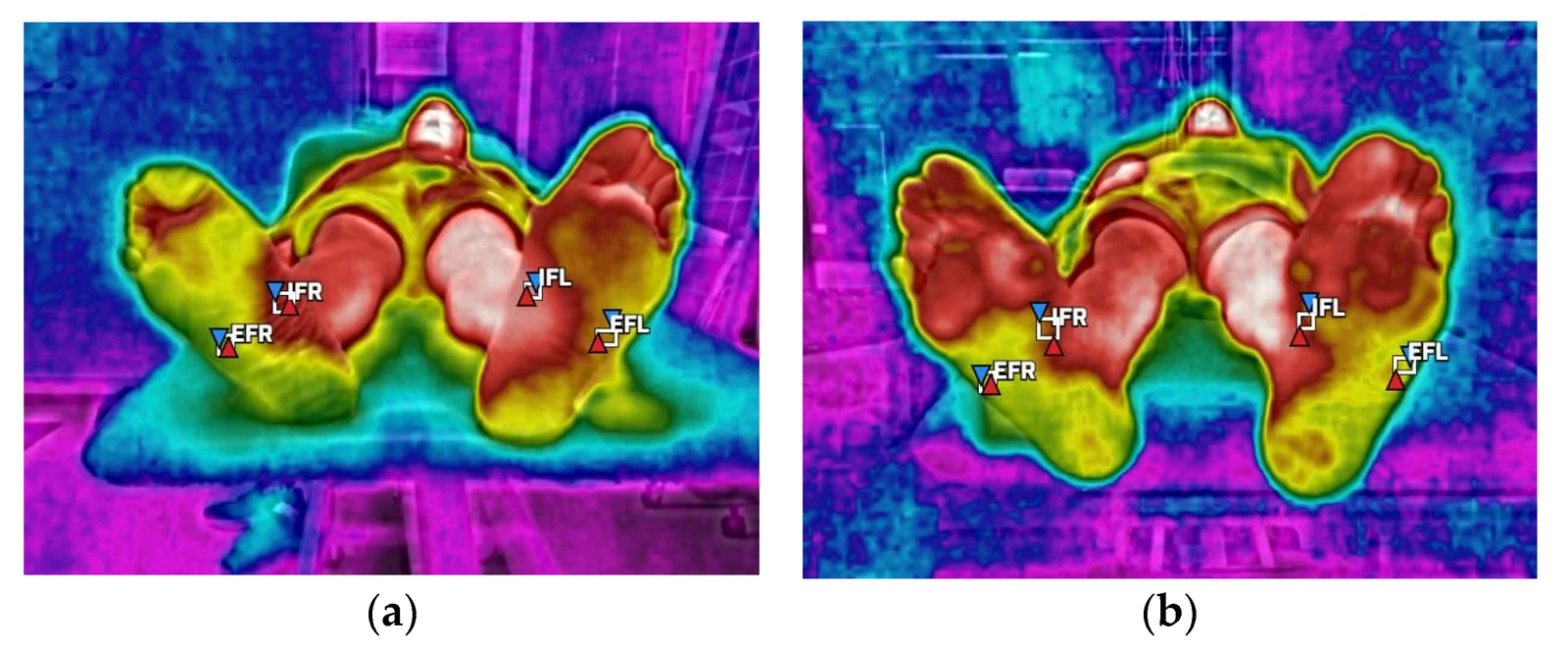
(**a**) Pre-intervention thermographic image showing plantar temperature distribution. (**b**) Post-intervention thermographic image showing plantar temperature distribution. IFR, internal foot right; IFL, internal foot left; EFR, external foot right; and EFL, external foot left.

Analysis of temperature differences between affected and unaffected limbs showed generalized reductions in thermal asymmetry after the intervention in both groups across anterior (Figure 2), posterior (Figure 3), and plantar regions (Figure 4), although with different degrees of consistency. The color scale represents skin temperature (°C), ranging from purple and dark blue (lowest temperatures), through light blue, green, and yellow (intermediate temperatures), to orange and red (highest temperatures). The triangular labels identify the anatomical location of each ROI.

Baseline asymmetry values ranged from 0.54 °C to 1.14 °C in the IG (Table 3) and from 0.61 °C to 1.10 °C in the CG (Table 4).

The IG exhibited a homogeneous pattern of asymmetrical reduction across all regions. Although most reductions did not reach statistical significance, several regions showed moderate to large effect sizes, suggesting meaningful changes in thermal symmetry. Reductions ranged from −0.11 °C to −0.50 °C, with more pronounced changes observed in the internal foot region (Δ = −0.50 °C; *p* = 0.054; r = 0.56) and the posterior inferior region (Δ = −0.39 °C; *p* = 0.058; r = 0.55), both corresponding to large effect sizes. A statistically significant reduction was observed in the posterior superior region, where asymmetry decreased from 0.99 °C to 0.64 °C (Δ = −0.35 °C; *p* = 0.013; r = 0.72), indicating a large effect size.

In the CG, thermal asymmetry also decreased in all regions, although none of the changes reached statistical significance. Reductions ranged from −0.14 °C to −0.49 °C. Nevertheless, moderate to large effect sizes were observed in several regions, particularly in the internal foot (Z = −1.924; *p* = 0.054; r = 0.61) and posterior inferior region (Z = −1.871; *p* = 0.061; r = 0.59). The largest absolute reduction was observed in the posterior superior region (Δ = −0.49 °C; Z = −1.247; *p* = 0.213; r = 0.39). Overall, changes in the CG were more heterogeneous than those observed in the IG.

### 3.6. Between-Group Comparison of Thermal Variables

Overall, no significant differences between groups were observed in thermal asymmetry, whereas differences emerged in the absolute change in skin temperature in specific anatomical regions.

### 3.7. Thermal Asymmetry Between Groups

Between-group comparison did not reveal statistically significant differences in the evolution of thermal asymmetry in any of the analyzed regions (*p* > 0.40 in all cases). Both the IG and CG exhibited similar reductions in thermal discrepancy between the affected and unaffected limbs, indicating similar changes in thermal symmetry following the respective interventions (Table 5).

### 3.8. Between-Group Comparison of Absolute Temperature Changes

In contrast to the findings for thermal asymmetry, analysis of absolute temperature changes revealed significant between-group differences in specific anatomical regions. The CG showed significantly greater temperature increases in the anterior distal region for both the affected (Z = −2.113; *p* = 0.035; r = 0.45) and unaffected limbs (Z = −2.081; *p* = 0.037; r = 0.44), as well as in the internal foot region for both the affected (Z = −2.210; *p* = 0.027; r = 0.47) and unaffected limbs (Z = −2.081; *p* = 0.045; r = 0.44). These differences were associated with moderate effect sizes.

A non-significant trend was observed in the anterior proximal region of the unaffected limb (Z = −1.882; *p* = 0.060; r = 0.40), also corresponding to a moderate effect size. No statistically significant differences were found in the remaining regions (*p* > 0.05), indicating comparable thermal responses between interventions across most anatomical areas (Table 6).

### 3.9. Baseline-Adjusted Between-Group Analyses

To account for baseline differences and provide adjusted between-group comparisons, ANCOVA analyses were performed using baseline values as covariates.

For lower-limb motor function, ANCOVA revealed a significant effect of group on post-intervention FMA-LE scores after adjustment for baseline values (F = 5.028, *p* = 0.037, ηp^2^ = 0.209), indicating superior motor recovery in the IG.

Similarly, for walking capacity, ANCOVA demonstrated a significant effect of group on post-intervention 6MWT performance after controlling for baseline walking distance (F = 11.750, *p* = 0.003, ηp^2^ = 0.382). Adjusted mean walking distance was higher in the IG than in the CG, confirming the superiority of treadmill training beyond baseline functional differences.

Regarding thermographic outcomes, most thermal asymmetry variables did not show significant adjusted between-group differences. However, posterosuperior thermal asymmetry remained significantly different between groups after adjustment for baseline values (F = 8.987, *p* = 0.007, ηp^2^ = 0.321), indicating a greater reduction in asymmetry in the IG (Table 7).

### 3.10. Relationship Between Thermal Asymmetry and Functional Recovery

Spearman correlation analyses were performed to explore the association between changes in thermal asymmetry and improvements in motor function (FMA-LE) and walking capacity (6MWT). No statistically significant correlations were identified between changes in any thermal asymmetry measure and changes in either FMA-LE or 6MWT (all *p* > 0.05). Correlation coefficients were consistently weak, suggesting that improvements in thermal symmetry occurred independently of improvements in functional performance.

### 3.11. Adverse Events

No adverse events were reported during the study.

## 4. Discussion

The present study examined the ability of infrared thermography to detect changes in thermal asymmetry following a 12-week physiotherapy program in individuals with chronic stroke, alongside changes in motor function and functional performance. The main finding was that infrared thermography identified intervention-induced changes, revealing reductions in thermal asymmetry between body sides in both groups after treatment. The primary hypothesis was only partially supported, as reductions in thermal asymmetry were observed following rehabilitation, but the addition of treadmill training did not produce consistent between-group differences across thermographic regions. In contrast, the secondary hypotheses were supported, with the IG showing greater improvements in motor function and walking capacity after adjustment for baseline values.

Furthermore, several thermographic regions exhibited moderate to large effect sizes, particularly in the internal foot and posterior lower-leg regions, suggesting potentially relevant reductions in thermal asymmetry even when statistical significance was not consistently achieved. These findings support the use of infrared thermography as a non-invasive tool [4] capable of detecting changes in thermal asymmetry associated with rehabilitation interventions after stroke, although an additional effect of treadmill training on thermographic outcomes was not consistently demonstrated.

From a physiological perspective, the changes observed in both groups (CG and IG) may reflect modifications in muscle activation and cutaneous perfusion, associated with exercise-induced peripheral adaptations [34]. The normalization of thermal symmetry suggests that thermography may capture physiological adaptations associated with musculoskeletal activation during physiotherapy, including changes in local blood flow and heat distribution in superficial tissues [35]. In this regard, the generalized and statistically significant pre–post increase in skin temperature across all analyzed regions, both proximal and distal, and in both the affected and unaffected sides [36] suggests that these thermal adaptations were not limited to specific areas but rather reflect a global neuromuscular activation [37] associated with the rehabilitation process.

Despite the changes detected by thermography in both groups, these thermal adaptations were not accompanied by equivalent improvements in motor function or functional performance. While reductions in thermal asymmetry were observed following rehabilitation regardless of group allocation, only participants who received additional treadmill training achieved significant gains in lower-limb motor function and walking capacity. Importantly, ANCOVA analyses confirmed that these differences remained significant even after adjusting for baseline values, indicating that the superiority of treadmill training cannot be explained solely by the higher initial functional status of the IG.

These findings suggest that thermal normalization and functional recovery may represent partially independent dimensions of the rehabilitation process. Conventional physiotherapy alone appeared sufficient to induce peripheral physiological adaptations reflected by increased skin temperature and reduced thermal asymmetry. However, these peripheral adaptations did not automatically translate into superior motor or functional outcomes. In contrast, the addition of treadmill training produced clear functional benefits while generating only limited additional changes in thermographic variables. This pattern suggests that infrared thermography may be capturing physiological processes related to peripheral regulation, tissue perfusion, metabolic activity, or generalized muscular activation, whereas functional recovery depends on additional mechanisms requiring task-specific, repetitive and intensive practice [33]. Consistent with this interpretation, no significant associations were observed between reductions in thermal asymmetry and improvements in FMA-LE or walking capacity. These findings suggest that thermographic changes may reflect physiological adaptations that are not directly captured by conventional functional assessments. Similar discrepancies between thermographic and functional responses have been reported previously, with improvements in motor performance occurring despite persistent thermal asymmetries [38]. This variability may reflect the complexity of peripheral thermal regulation after stroke, as well as differences in intervention characteristics, assessment timing, and thermographic protocols, as highlighted in a recent systematic review [6].

An interesting finding is that most thermographic variables lost their between-group differences after baseline-adjusted analyses, whereas functional outcomes remained significantly different between groups. Only posterosuperior thermal asymmetry maintained statistical significance after adjustment. Taken together, these results reinforce the idea that thermographic adaptations are largely induced by rehabilitation itself, independently of the specific training modality applied. Conversely, the superior functional outcomes observed in the treadmill group appear to reflect the additional contribution of task-oriented gait practice and the greater overall training exposure received by the IG.

In this regard, the conventional physiotherapy applied primarily focused on training capacities such as strength, mobility, and balance, as well as closed kinetic chain tasks—without a specific emphasis on the repetition of the functional task of walking [39,40], which may have been insufficient to promote transfer to functional performance.

The functional improvements observed in the IG may be primarily explained by the greater intensity, increased practice volume, and high repetition of gait cycles associated with treadmill training [41]. These characteristics are consistent with principles of task-specific motor learning and may have promoted more symmetrical activation, improved selective control of the affected lower limb, and enhanced the capacity to sustain prolonged walking efforts. The persistence of significant between-group differences after baseline adjustment further supports the contribution of treadmill training beyond pre-existing functional differences between groups. These findings are in line with previous evidence showing that high-intensity treadmill training is more effective than lower-intensity approaches for improving walking capacity and motor performance in individuals with chronic stroke, supporting its use as an effective adjunct to conventional rehabilitation [40,41].

From a clinical perspective, the ability to sustain walking during submaximal efforts is a key determinant of functional capacity and long-term prognosis after stroke [42], making the observed improvement in distance covered during the 6MWT potentially meaningful. Participants in the IG increased their walking distance by approximately 64 m after the 12-week intervention, accompanied by a large within-group effect size. This improvement is particularly relevant from a clinical perspective, as its magnitude compares favorably with previously proposed thresholds for meaningful change in the 6MWT after stroke. Some authors reported a meaningful change of approximately 34.4 m in individuals with chronic stroke [43], while more recent evidence has considered improvements of 20 m and 50 m as indicative of small and moderate clinically important changes [44]. In this context, the improvement observed in the present study not only exceeded these proposed thresholds but also surpassed the level considered indicative of a moderate clinically important change, supporting the clinical relevance of the gains in walking endurance beyond their statistical significance.

This finding is particularly noteworthy considering the chronic stage of the participants, in which limitations in walking capacity frequently persist and walking recovery remains an important rehabilitation priority. The magnitude of improvement observed in the IG is also consistent with evidence supporting intensive and task-specific locomotor training for improving walking capacity after stroke [40,45]. In contrast, the CG did not show a comparable improvement in walking performance, further supporting the added functional benefit of treadmill training observed in the present study.

Regarding motor function, the significant improvement observed in the FMA-LE in the IG, together with the large effect size and the persistence of significant between-group differences after baseline-adjusted ANCOVA, reinforces the functional relevance of the intervention. Improvements in lower-limb motor control are closely associated with enhanced gait performance and functional independence after stroke [11], reinforcing the potential value of treadmill training during chronic rehabilitation.

In the context of chronic stroke, where reduced functional endurance is associated with greater dependence and poorer functional outcomes [46,47], these findings support the value of interventions specifically aimed at improving walking endurance as a priority target of rehabilitation in this phase [48,49]. Furthermore, these improvements may be associated with an increase in daily step count and a reduction in sedentary time [50], with potentially important implications for participation and quality of life.

In addition, these changes may be mediated by integrated neuromuscular and cardiorespiratory adaptations [41], such as improved muscle efficiency, optimized oxygen utilization during submaximal exercise, and a more efficient organization of gait patterns. However, these mechanisms should be interpreted as plausible, as they were not directly assessed in the present study.

Therefore, infrared thermography should not be considered a surrogate marker of functional recovery. Instead, it may provide complementary information regarding peripheral physiological adaptations that occur during rehabilitation [4,13], including thermal changes that may not be reflected by parallel changes in motor or functional performance. The observed discrepancy between thermal normalization and functional improvement may represent one of the most relevant findings of the present study, suggesting that recovery after stroke involves multiple dimensions that do not necessarily evolve in parallel. While thermography appears to capture peripheral adaptations potentially related to muscle activation and tissue perfusion induced by treatment, improvements in motor function and walking capacity during prolonged efforts likely depend on more complex processes of neuromuscular integration and locomotor pattern reorganization.

### Study Limitations and Future Directions

The present study has several limitations that should be considered when interpreting the results.

First, baseline differences between groups must be acknowledged. Although participants were randomly allocated, a significant baseline difference was observed in functional walking capacity, with the IG demonstrating higher 6MWT performance than the CG before treatment. In addition, some variability in baseline thermal asymmetry values was observed across anatomical regions. To address this potential source of bias, both change-score analyses and baseline-adjusted ANCOVA models were performed.

The relatively small sample size may also have limited the ability to detect significant between-group differences in some thermographic variables, particularly in regions showing moderate to large effect sizes despite non-significant *p*-values.

Although thermal asymmetry was the primary outcome, no previous intervention studies were available to estimate the expected effect size for thermographic measures or the extended FMA-LE protocol. Therefore, the sample size calculation was based on the validated FMA-LE motor subscale (0–34), which represented the best available evidence. Despite being a sensitive tool for assessing variations in cutaneous perfusion and thermal symmetry, infrared thermography may be influenced by factors such as thermal fluctuations occurring prior to assessment. Moreover, the absence of complementary physiological measures, such as cardiorespiratory parameters or biomechanical analysis, limits the ability to more precisely link thermal changes to the neurophysiological mechanisms underlying motor and aerobic adaptations. Furthermore, manual ROI segmentation, despite being performed by a single blinded evaluator following standardized anatomical references, may have introduced observer-dependent variability, although a consistent protocol was applied across all thermograms.

Another limitation was the unequal treatment exposure between groups, as treadmill training was added to conventional physiotherapy rather than replacing an equivalent amount of therapy. Consequently, the IG received a greater overall rehabilitation dose, and the observed functional benefits cannot be attributed exclusively to treadmill-specific effects. The additional rehabilitation exposure may also have contributed to the observed between-group differences.

Multiple comparisons were performed across several predefined thermographic regions without adjustment for multiple testing. Accordingly, the regional thermographic analyses should be considered exploratory, and individual region-specific findings should be interpreted with caution.

Finally, the lack of longitudinal follow-up prevents determination of the persistence of the observed improvements and whether maintenance programmes are required to ensure their long-term stability.

Future research should include larger samples and longitudinal designs to increase statistical power, enhance the generalizability of the findings and examine the temporal evolution of training-induced thermal and aerobic adaptations. The implementation of protocols with clearly defined progression phases could help clarify whether these adaptations follow distinct trajectories or converge at more advanced stages of rehabilitation.

Likewise, the integration of multimodal physiological measures, such as cardiorespiratory parameters, electromyography and kinematic–biomechanical analysis, would allow for a more precise characterization of the neurophysiological mechanisms underlying the observed functional and thermal changes. In this context, the application of artificial intelligence and deep learning techniques to the analysis of thermal images emerges as a particularly promising avenue for objective feature extraction and the development of predictive models of functional recovery, with potential clinical application in neurological rehabilitation.

## 5. Conclusions

Infrared thermography enabled the detection of changes in thermal asymmetry of the lower limbs following intervention in individuals with chronic stroke. In addition, the incorporation of treadmill training was associated with improvements in lower-limb motor function and distance covered.

However, these thermographic adaptations showed a different pattern of change from that observed for motor function and walking performance. While thermal asymmetry decreased in both groups, significant functional improvements were observed only in participants who received additional treadmill training.

Taken together, these findings support the role of thermography as a supplementary assessment tool in stroke rehabilitation. The differing patterns observed between thermographic and functional outcomes suggest that these assessments provide complementary information on recovery and should therefore be used alongside established clinical outcome measures.

## Figures and Tables

**Table 1:** Baseline descriptive values of FMA-LE, 6MWT, and regional temperatures by groups. Abbreviations: IG, intervention group; CG, control group; PRE, pre-intervention; FMA-LE, Fugl Meyer Assessment—Lower Extremity; 6MWT, Six-Minute Walk Test.

Variable	IG (n = 12) M ± Sd	CG (n = 10) M ± Sd	Value
FMA-LE_PRE	73.58 ± 9.05	67.20 ± 13.35	0.381
SixMWT_PRE (m)	373.51 ± 115.76	222.61 ± 131.95	0.014
PRE-Affected AnteroInferior (°C)	28.62 ± 2.45	27.75 ± 1.90	0.283
PRE-Non-Affected AnteroInferior (°C)	29.49 ± 2.22	28.52 ± 1.98	0.346
PRE-Affected AnteroSuperior (°C)	28.06 ± 2.29	27.82 ± 1.78	0.974
PRE-Non-Affected AnteroSuperior(°C)	28.57 ± 2.20	27.92 ± 2.09	0.582
PRE-Affected External Foot (°C)	24.44 ± 2.05	25.06 ± 2.47	0.456
PRE-Non-Affected External Foot (°C)	25.09 ± 1.56	25.05 ± 2.11	0.722
PRE-Affected- Internal Foot (°C)	28.16 ± 1.55	27.64 ± 2.84	0.872
PRE-Non-Affected Internal Foot (°C)	28.83 ± 1.51	27.84 ± 2.31	0.381
PRE-Affected PosteroInferior (°C)	26.85 ± 2.39	26.59 ± 2.18	0.722
PRE-Non-Affected PosteroInferior (°C)	27.63 ± 2.15	27.38 ± 1.83	0.582
PRE-Affected PosteroSuperior (°C)	29.46 ± 2.04	29.19 ± 1.26	0.821
PRE-Non-Affected PosteroSuperior (°C)	29.95 ± 1.97	29.56 ± 1.13	0.381

**Table 2:** Pre- and post-intervention temperatures by anatomical region and by group (°C). Abbreviations: IG, intervention group; CG, control group; PRE, pre-intervention; POST, post-intervention; *p*, statistical significance; ** *p* < 0.01; * *p* < 0.05.

Region	IG–PRE Affected	IG–POST Affected	IG–PRE Non-Affected	IG–POST Non-Affected	CG–PRE Affected	CG–POST Affected	CG–PRE Non-Affected	CG–POST Non-Affected
AnteroInferior	28.6 ± 2.4	31.1 ± 1.0	29.5 ± 2.2	31.4 ± 1.2	27.8 ± 1.9	31.3 ± 1.0	28.5 ± 2.0	31.6 ± 1.0
*p*	0.008 **		0.011 *		0.008 **		0.008 **	
AnteroSuperior	28.1 ± 2.3	30.7 ± 1.6	28.6 ± 2.2	31.0 ± 1.5	27.8 ± 1.8	31.0 ± 1.2	27.9 ± 2.1	31.3 ± 1.3
*p*	0.003 **		0.008 **		0.008 **		0.008 **	
External Foot	24.4 ± 2.1	26.8 ± 2.0	25.1 ± 1.6	27.3 ± 2.0	25.1 ± 2.5	28.0 ± 1.3	25.1 ± 2.1	28.3 ± 1.7
*p*	0.005 **		0.008 **		0.008 **		0.008 **	
Internal Foot	28.2 ± 1.5	29.9 ± 1.7	28.8 ± 1.5	30.2 ± 1.8	27.6 ± 2.8	30.3 ± 1.9	27.8 ± 2.3	30.6 ± 1.3
*p*	0.007 **		0.007 **		0.011 *		0.008 **	
PosteroInferior	26.9 ± 2.4	29.1 ± 2.2	27.6 ± 2.1	29.7 ± 2.4	26.6 ± 2.2	29.8 ± 1.4	27.4 ± 1.8	30.3 ± 1.2
*p*	0.005 **		0.005 **		0.008 **		0.008 **	
PosteroSuperior	29.5 ± 2.0	30.8 ± 1.4	29.9 ± 2.0	31.2 ± 1.8	29.2 ± 1.3	31.7 ± 0.9	29.6 ± 1.1	31.7 ± 0.9
*p*	0.017 *		0.011 *		0.008 **		0.008 **	

**Table 3:** Pre–post thermal asymmetries, absolute change, and statistical significance. Abbreviations: IG, intervention group; PRE, pre-intervention; POST, post-intervention.

Region	IG PRE	IG POST	Δ IG	Z	*p*	r
AnteroInferior	1.14 ± 1.37	0.73 ± 0.58	–0.41	–1.090	0.276	0.31
AnteroSuperior	0.54 ± 0.71	0.43 ± 0.29	–0.11	–1.409	0.159	0.41
External Foot	0.80 ± 0.67	0.54 ± 0.32	–0.26	–1.439	0.150	0.42
Internal Foot	1.02 ± 0.82	0.52 ± 0.39	–0.50	–1.924	0.054	0.56
PosteroInferior	1.11 ± 1.02	0.72 ± 0.57	–0.39	–1.895	0.058	0.55
PosteroSuperior	0.99 ± 1.10	0.64 ± 0.47	–0.35	–2.488	0.013	0.72

**Table 4:** Pre–post thermal asymmetries, absolute change, and statistical significance. Abbreviations: CG, control group; PRE, pre-intervention; POST, post-intervention.

Region	CG PRE	CG POST	Δ CG	Z	*p*	r
AnteroInferior	0.87 ± 0.68	0.65 ± 0.43	–0.22	–0.747	0.455	0.24
AnteroSuperior	0.90 ± 0.79	0.44 ± 0.29	–0.46	–0.311	0.755	0.1
External Foot	0.71 ± 0.70	0.48 ± 0.55	–0.23	–1.336	0.181	0.42
Internal Foot	1.10 ± 1.07	0.76 ± 1.06	–0.34	–1.924	0.054	0.61
PosteroInferior	0.79 ± 0.62	0.65 ± 0.50	–0.14	–1.871	0.061	0.59
PosteroSuperior	0.61 ± 0.60	0.12 ± 0.29	–0.49	–1.247	0.213	0.39

**Table 5:** Differences in thermal asymmetry. Abbreviations: DIF_Asim, difference in thermal asymmetries.

Variable	U	Z	*p*	r
DIF_Asim-AnteroInferior	55.50	–0.300	0.764	0.06
DIF_Asim_AnteroSuperior	47.50	–0.827	0.408	0.18
DIF_Asim_External Foot	44.50	–1.026	0.305	0.22
DIF_Asim_Internal Foot	47.50	–0.827	0.408	0.18
DIF_Asim_PosteroInferior	52.00	–0.532	0.595	0.11
DIF_Asim_PosteroSuperior	58.00	–0.133	0.894	0.03

**Table 6:** Differences in absolute temperature change. Abbreviations: Dif, temperature difference.

Variable	U	Z	*p*	r
Dif_Afeccted_AnteroInferior	28.00	–2.113	0.035	0.45
Dif_Non-Affected_AnteroInferior	28.50	–2.081	0.037	0.44
DIF_Affected_AnteroSuperior	41.50	–1.222	0.222	0.26
Dif_Non-Affected_AnteroSuperior	31.50	–1.882	0.060	0.4
Dif_Affected_External Foot	28.50	–2.081	0.037	0.44
Dif_Non-Affected_External Foot	41.50	–1.222	0.222	0.26
Dif_Affected_Internal Foot	31.00	–2.210	0.027	0.47
Dif_Non-Affected_Internal Foot	31.50	–2.081	0.045	0.44
Dif_Affected_PosteroInferior	41.50	–1.222	0.222	0.26
Dif_Non-Affected_PosteroInferior	41.50	–1.222	0.222	0.26
Dif_Affected_PosteroSuperior	55.50	–0.300	0.764	0.06
Dif_Non-Affected_PosteroSuperior	58.00	–0.133	0.894	0.03

**Table 7:** Analysis of covariance (ANCOVA) comparing post-intervention outcomes between groups after adjustment for baseline values. Abbreviations: ANCOVA, analysis of covariance; FMA-LE, Fugl–Meyer Assessment—Lower Extremity; 6MWT, Six-Minute Walk Test; η^2^, partial eta squared.

Outcome Variable	Covariate	F	*p*-Value	Partial η^2^	Interpretation
FMA-LE POST	Baseline FMA-LE	5.028	0.037	0.209	Significant group effect
6MWT POST	Baseline 6MWT	11.750	0.003	0.382	Significant group effect
Thermal asymmetry–Anteroinferior	Baseline anteroinferior asymmetry	0.013	>0.05	—	No significant group effect
Thermal asymmetry–Anterosuperior	Baseline anterosuperior asymmetry	0.065	>0.05	—	No significant group effect
Thermal asymmetry–External Foot	Baseline external foot asymmetry	0.156	>0.05	0.008	No significant group effect
Thermal asymmetry–Internal Foot	Baseline internal foot asymmetry	0.480	>0.05	—	No significant group effect
Thermal asymmetry–Posteroinferior	Baseline posteroinferior asymmetry	0.214	>0.05	—	No significant group effect
Thermal asymmetry–Posterosuperior	Baseline posterosuperior asymmetry	8.987	0.007	0.321	Significant group effect

## Data Availability

The datasets generated and analyzed during the current study are publicly available in Mendeley Data: https://data.mendeley.com/datasets/d2f2vmts78/1 (accessed on 1 June 2026).

## Abbreviations

The following abbreviations are used in this manuscript:

CONSORT	Consolidated Standards of Reporting Trials
TIDieR	Template for Intervention Description and Replication
FMA-LE	Fugl–Meyer Assessment for the Lower Extremity
6MWT	Six-Minute Walk Test
TISEM	Thermographic Imaging in Sports and Exercise Medicine
ROI	Region of Interest
IG	Intervention Group
CG	Control Group
